# Anti-SARS-CoV-2 Spike Antibody Titers and Neutralizing Antibodies in Vaccinated Rheumatoid Arthritis Patients

**DOI:** 10.3390/vaccines10081365

**Published:** 2022-08-21

**Authors:** Hiroshi Furukawa, Shomi Oka, Takashi Higuchi, Moriyuki Nakama, Nobuhiro Nagai, Shigeto Tohma

**Affiliations:** 1 Department of Rheumatology, National Hospital Organization Tokyo National Hospital, 3-1-1 Takeoka, Kiyose 204-8585, Japan; 2 Department of Nephrology, Ushiku Aiwa General Hospital, 896 Shishiko-cho, Ushiku 300-1296, Japan; 3 Department of Clinical Laboratory, National Hospital Organization Tokyo National Hospital, 3-1-1 Takeoka, Kiyose 204-8585, Japan; 4 Department of Clinical Laboratory, National Hospital Organization Shimofusa Psychiatric Medical Center, 578 Heta-cho, Midori-ku, Chiba 266-0007, Japan

**Keywords:** vaccination, anti-SARS-CoV-2 spike antibody, anti-SARS-CoV-2 neutralizing antibody, rheumatoid arthritis

## Abstract

Coronavirus disease 2019 (COVID-19) is caused by severe acute respiratory syndrome coronavirus 2 (SARS-CoV-2). A serological test is used to assess the efficacy of vaccination. It has been reported that anti-SARS-CoV-2 spike (S) and neutralizing antibody (Ab) levels are lower following vaccination in patients with rheumatic disease. Here, we investigated anti-SARS-CoV-2 S and neutralizing Abs in vaccinated rheumatoid arthritis (RA) patients in Japan. Anti-SARS-CoV-2 S and neutralizing Abs were quantified in 101 RA patients and 117 controls. Anti-SARS-CoV-2 S Ab levels were lower in RA patients than both earlier after vaccination in controls (mean RA 324.1 ± 591.8 SDM vs. control 1216.6 ± 854.4 [U/mL], *p* < 0.0001) and later after vaccination (324.1 ± 591.8 vs. 582.0 ± 415.6 [U/mL], *p* = 0.0002). The interval between vaccination of the RA patients and serum collection was longer than for controls early after vaccination (142.1 ± 31.6 vs. 98.3 ± 11.2 [days], *p* < 0.0001), but shorter than the later sample from the controls (142.1 ± 31.6 vs. 257.3 ± 11.2 [days], *p* < 0.0001). Importantly, anti-SARS-CoV-2 neutralizing Ab titers in RA patients were higher than in either early or later control samples (10.7 ± 4.9 vs. 8.6 ± 6.6 [%], *p* = 0.0072, and 10.7 ± 4.9 vs. 3.1 ± 3.7 [%], *p* < 0.0001, respectively). Anti-SARS-CoV-2 S Ab titers in vaccinated RA patients were lower than in controls, but they were influenced by other clinical manifestations. Anti-SARS-CoV-2 neutralizing Ab levels were independently increased in RA.

## 1. Introduction

The outbreak of coronavirus disease 2019 (COVID-19) was first reported in Wuhan [1], found to be caused by severe acute respiratory syndrome coronavirus 2 (SARS-CoV-2). For diagnosis, a quantitative reverse transcription polymerase chain reaction from sputum, nasal swab, or saliva samples is commonly performed. The presence of anti-SARS-CoV-2 spike (S) antibody (Ab) is investigated as a surrogate for the efficacy of vaccination and anti-SARS-CoV-2 nucleocapsid (N) Ab is detected to establish prior SARS-CoV-2 infection. Neutralizing Ab is measured to evaluate the protective capacity of serum Abs against SARS-CoV-2 infection. Plaque-reduction neutralization tests (PRNT) using SARS-CoV-2 and enzyme-linked immuno-sorbent assay (ELISA)-based neutralizing Ab assays are used for the detection of neutralizing Ab; the results of these two assays correlate well [2,3,4]. It has been reported that anti-SARS-CoV-2 S Ab titers persisted longer [5,6,7] than neutralizing Ab [8] after vaccination. Anti-SARS-CoV-2 S and neutralizing Ab levels were reported to be lower in patients with rheumatic diseases, including rheumatoid arthritis (RA), with anti-SARS-CoV-2 S Ab even being undetectable in some of these patients after vaccination [9,10,11,12,13]. However, anti-SARS-CoV-2 S titers and neutralizing Abs have not been extensively investigated in vaccinated RA patients. Hence, here, we measured anti-SARS-CoV-2 S and neutralizing Abs in vaccinated RA patients in Japan.

## 2. Materials and Methods

### 2.1. Patients and Sera 

A total of 101 RA patients was recruited at Tokyo National Hospital. The RA patients fulfilled Rheumatoid Arthritis Classification Criteria [14] or American College of Rheumatology Criteria for RA [15]. None of the RA patients were diagnosed with COVID-19 before the collection of sera post vaccination. All were vaccinated twice against SARS-CoV-2 with BNT162b2 (Pfizer, New York, NY, USA) or mRNA-1273 (Moderna, Cambridge, MA, USA). Two of the RA patients were vaccinated with mRNA-1273, 57 were vaccinated with BNT162b2, and the information of the others was not available. Sera were collected from RA patients before the third vaccination. A total of 117 control subjects was recruited from healthcare workers at Tokyo National Hospital, twice vaccinated with BNT162b2. As with the RA patients, none of the controls were diagnosed with COVID-19 before serum collection. Sera were collected twice for health checkup (early and late collection) from the controls at different times before their third vaccination. 

The study was reviewed and approved by the Research Ethics Committee of Tokyo National Hospital (469). Written informed consent was obtained from all RA patients and controls. This study was conducted in accordance with the principles expressed in the Declaration of Helsinki. 

### 2.2. Detection of Anti-SARS-CoV-2 N, S, and Neutralizing Abs

Anti-SARS-CoV-2 N Ab titers were analyzed using chemiluminescent enzyme im-munoassays (Anti-SARS-CoV-2 N IgG, Fujirebio, Hachioji, Japan, User’s manual 2021-09 V1.0, P8B01T). The IgG fraction of anti-SARS-CoV-2 N Abs was measured in the assay. The cut-off value was 1.0 U/mL. Anti-SARS-CoV-2 S Ab titers were quantified by the electrochemiluminescence immunoassay system Elecsys Anti-SARS-CoV-2 S (Roche Diagnostics, Mannheim, Germany, Package Insert 2020-09, V1.0, #09289275190). The Abs including IgG to the receptor binding domain on S1 subunit (Wuhan-Hu-1) were measured in the assay. The cut-off value was 0.8 U/mL. Anti-SARS-CoV-2 neutralizing Abs were measured by the blocking ELISA system SARS-CoV-2 Neutralization Antibody Detection Kit (Medical & Biological Laboratories Co., Ltd., Tokyo, Japan, User’s manual version #201015, https://ruo.mbl.co.jp/bio/dtl/dtlfiles/5360_v201015.pdf accessed on 10 August 2022) [16]. The inhibition effects of anti-SARS-CoV-2 Abs blocking the interaction of receptor-binding domain (Wuhan-Hu-1) and angiotensin converting enzyme 2 were evaluated in the assay. This assay and PRNT were correlated (https://ruo.mbl.co.jp/bio/product/sars-cov-2/pickup/SARS-CoV-2-neutralizing-antibody.html). The inhibition rate was calculated as follows: inhibition rate = (1 − optical density value of sample/optical density value of blank) × 100 (%). The cut-off value was 11.68% based on the 98th percentile among 52 pre-pandemic control subjects, as previously reported [7]. Assays for SARS-CoV-2 Abs were not performed repeatedly. The results of anti-SARS-CoV-2 N, S, and neutralizing Ab for some of the controls were previously reported [7].

### 2.3. Statistical Analysis

Differences in demographic features and experimental findings of RA patients were analyzed by Fisher’s exact test using 2 × 2 contingency tables or Student’s *t*-test. Simple linear regression analysis was conducted to estimate whether clinical characteristics correlated with anti-SARS-CoV-2 S titers or neutralizing Abs in RA patients. Deviation from zero was tested for partial regression coefficients (PRC); *p* values were calculated. Clinical characteristics with a PRC > 0 associated with increased anti-SARS-CoV-2 S or neutralizing Ab in RA patients and those with PRC < 0 with a decrease. Multiple linear regression analysis of a clinical characteristic was conducted on the results from RA patients and controls (early collection) to identify the independent association of the clinical characteristic from the others. 

## 3. Results

### 3.1. Characteristics of the RA Patients and Controls

Clinical manifestations of the RA patients and controls are described in Table 1. RA patients were older than controls. 

### 3.2. Anti-SARS-CoV-2 Abs in Sera of RA Patients

Anti-SARS-CoV-2 Ab titers were quantified in the RA patients (Table 2). Anti-SARS-CoV-2 N Abs were not detected in any RA patients or controls (late collection). S Ab was detected in almost all RA patients, but the levels were lower than they were in controls, both early and later after vaccination (324.1 ± 591.8 vs. 1216.6 ± 854.4 (U/mL), *p* < 0.0001 and 324.1 ± 591.8 vs. 582.0 ± 415.6 (U/mL), *p* = 0.0002, respectively). However, the interval between vaccination and serum collection of the RA patients was longer than the earlier samples from the controls (142.1 ± 31.6 vs. 98.3 ± 11.2 (days), *p* < 0.0001), but shorter than the later time point (142.1 ± 31.6 vs. 257.3 ± 11.2 (days), *p* < 0.0001). The percentage of RA patients positive for anti-SARS-CoV-2 S Ab was slightly lower than controls, both early (*n* = 97 (96.0%) vs. *n* = 116 (100.0%), *p* = 0.0454) or later (vs. *n* = 116 (100.0%), *p* = 0.0454) after vaccination. Thus, a minority of RA patients was unable to raise anti-SARS-CoV-2 S Ab at all after vaccination.

In contrast, anti-SARS-CoV-2 neutralizing Ab was present in RA patients after vaccination, remarkably, at levels higher than in either early samples from controls (10.7 ± 4.9 vs. 8.6 ± 6.6 (%), *p* = 0.0072) or later samples (10.7 ± 4.9 vs. 3.1 ± 3.7 (%), *p* < 0.0001). The proportion of RA patients with anti-SARS-CoV-2 neutralizing Ab tended to be higher than in controls earlier after vaccination (*n* = 40 (39.6%) vs. *n* = 33 (28.4%), *p* = 0.0865) and this difference became significant later (*n* = 40 (39.6%) vs. *n* = 5 (4.3%), *p* < 0.0001). Thus, anti-SARS-CoV-2 S Ab titers were lower in the RA patients, but anti-SARS-CoV-2 neutralizing Ab levels and the proportion of patients possessing such Abs were higher.

### 3.3. Clinical Correlations with Anti-SARS-CoV-2 Ab

Simple linear regression analysis was conducted to seek influences of any clinical manifestations on anti-SARS-CoV-2 Ab in the RA patients (Table 3). Age was found to be negatively associated with anti-SARS-CoV-2 S Ab level (PRC −18.20, 95%CI −28.47~−7.92, *p* = 0.0007). The interval between the last vaccination and serum collection was also associated with the anti-SARS-CoV-2 S Ab level (PRC 4.21, 95%CI 0.62~7.80, *p* = 0.0222). Rheumatoid factor was negatively associated with anti-SARS-CoV-2 neutralizing Ab level (PRC −0.0003, 95%CI −0.0006~0.0000, *p* = 0.0390). Biological disease-modifying anti-rheumatic drug administration was also negatively associated with anti-SARS-CoV-2 neutralizing Ab level (PRC −0.38, 95%CI −0.65~−0.10, *p* = 0.0071). Thus, some clinical manifestations were found be associated with the anti-SARS-CoV-2 Ab level after mRNA vaccination.

Age was negatively associated with anti-SARS-CoV-2 S Ab level in controls, both early (PRC −28.39, 95%CI −39.84~−16.94, *p* < 0.0001, Supplementary Table S1) or later (PRC −12.35, 95%CI −18.04~−6.67, *p* < 0.0001) after vaccination. Male sex was also negatively associated with anti-SARS-CoV-2 S Ab level in controls, both early (PRC −420.38, 95%CI −745.24~−95.52, *p* = 0.0117) and later (PRC −167.12, 95%CI −326.64~−7.60, *p* = 0.0402) after vaccination. The interval between the last vaccination and serum collection was negatively associated with the anti-SARS-CoV-2 S Ab level in the earlier samples from the controls (PRC −14.11, 95%CI −28.01~−0.21, *p* = 0.0467). The interval between the last vaccination and serum collection was negatively associated with the anti-SARS-CoV-2 neutralizing Ab level in the later samples from the controls (PRC −0.07, 95%CI −0.13~−0.01, *p* = 0.0318). Thus, some clinical features were associated with the anti-SARS-CoV-2 Ab level after vaccination in controls, though the manner of associations was not the same.

Multiple linear regression analysis for anti-SARS-CoV-2 S and neutralizing Ab was conducted to identify the independent association of clinical manifestations (Supplementary Table S2). The significant negative association of age with anti-SARS-CoV-2 S Ab was detected, when conditioned on the other clinical manifestations (PRC_adjusted_ −22.86, 95%CI −31.02~−14.71, *p*_adjusted_ < 0.0001). The interval between the last vaccination and serum collection was negatively associated with the anti-SARS-CoV-2 neutralizing Ab level (PRC_adjusted_ −0.05, 95%CI −0.09~−0.02, *p*_adjusted_ =0.0022). The association of RA with the anti-SARS-CoV-2 neutralizing Ab level still remained significant (PRC_adjusted_ 5.48, 95%CI 2.44~8.52, *p*_adjusted_ = 0.0005) when conditioned, suggesting an independent association. Thus, some clinical manifestations were independently associated with the anti-SARS-CoV-2 Ab level after vaccination.

## 4. Discussion

In the present study, anti-SARS-CoV-2 S Ab levels were found to be lower in RA patients than in controls after mRNA vaccination. That anti-SARS-CoV-2 S Ab levels are lower in patients with rheumatic diseases has been reported before and even that anti-SARS-CoV-2 S Ab are not produced at all in some patients after vaccination [9,10,11,12,13]. Our results are consistent with those of previous studies, though the significant association disappeared when conditioned. However, here, we found that anti-SARS-CoV-2 neutralizing Ab levels in RA patients were apparently higher than in controls, in contrast to previous studies. This association remained significant when conditioned, suggesting an independent association.

It is possible that the results of anti-SARS-CoV-2 neutralizing Ab assays could have been influenced by certain factors other than rheumatoid factor, which might have interfered with the assay, because no positive association between rheumatoid factor and anti-SARS-CoV-2 neutralizing Ab was detected in linear regression analysis (Table 3). It has been reported that anti-rat immunoglobulin light-chain Abs found in human sera might interfere with the ELISA assay [17], which was also suspected for immunochromatographic assays [18]. These results suggest that similar interference mechanisms may result in the apparently increased anti-SARS-CoV-2 neutralizing Ab levels detected in RA patients by the kit used in the present study.

No patient or control was diagnosed with COVID-19 before serum collection. Anti-SARS-CoV-2 N Abs were not detected in any RA patients or controls (late collection) [7]. However, it was reported that anti-SARS-CoV-2 N Ab was not found in about 15% of COVID-19 patients after 14 days of infection [19]. Thus, a different ratio of subclinical infection of COVID-19 in RA patients and controls could cause the higher levels of anti-SARS-CoV-2 neutralizing Ab in RA, though it cannot explain the lower levels of anti-SARS-CoV-2 S Ab in RA. 

Anti-SARS-CoV-2 neutralizing Ab levels in subjects vaccinated with mRNA-1273 were higher than those with BNT162b2 [20,21]. In the present study, RA patients were vaccinated with BNT162b2 or mRNA-1273, though controls were vaccinated with BNT162b2. Further, 3% of RA patients were vaccinated with mRNA-1273, while the information of vaccination was obtained in less than 60% of RA patients. Thus, it is possible that the difference between vaccines might cause the higher anti-SARS-CoV-2 neutralizing Ab levels in RA, though it cannot explain the lower levels of anti-SARS-CoV-2 S Ab in RA.

Apart from that, this report on anti-SARS-CoV-2 S and neutralizing Ab in vaccinated RA has some other limitations. The sample size is modest. Larger-scale independent studies need to be performed. The results of anti-SARS-CoV-2 neutralizing Abs in patients with RA and other autoimmune diseases should also be validated by several methods, including PRNT or chemiluminescent immunoassay, for neutralizing Ab. The mechanisms responsible for the apparently increased anti-SARS-CoV-2 neutralizing Ab found in RA patients should be clarified, as discussed above. The role of memory B and memory T cells should be investigated to evaluate the immunological function in RA patients after vaccination [11,12,22]. 

## 5. Conclusions

In the present study, anti-SARS-CoV-2 S Ab levels were decreased in RA patients but were influenced by other clinical manifestations. Anti-SARS-CoV-2 neutralizing Ab levels were independently increased in RA compared with controls, suggesting an influence of RA on anti-SARS-CoV-2 neutralizing Ab levels after vaccination. However, independent replication studies need to be performed; the results of anti-SARS-CoV-2 neutralizing Abs in RA patients should also be validated by other methods.

## Figures and Tables

**Table 1:** Characteristics of RA patients and controls.

	RA	Controls	*p*
Number	101	117	
Age, years (SD)	71.3 (10.7)	39.5 (12.6)	<0.0001
Male, *n* (%)	23 (22.8)	39 (33.6)	* 0.0974
Age at onset, years (SD)	60.4 (16.1)		
Steinbrocker stage III and IV, *n* (%)	36 (36.0)		
Steinbrocker class 3 and 4, *n* (%)	14 (14.0)		
Body mass index, kg/m^2^ (SD)	21.4 (3.9)		
Smoker or past smoker, *n* (%)	35 (35.7)		
RF, IU/mL (SD)	179.1 (338.8)		
ACPA, IU/mL (SD)	199.8 (249.2)		
DAS28 (SD)	3.0 (0.8)		
DAS28-CRP (SD)	2.0 (0.7)		
Corticosteroid administration, *n* (%)	38 (37.6)		
csDMARDs administration, *n* (%)	67 (66.3)		
bDMARDs administration, *n* (%)	14 (13.9)		
tsDMARDs administration, *n* (%)	25 (24.8)		

Numbers or average values for each group are shown. Standard deviations or percentages are shown in parentheses. Significance of differences was tested by Student’s *t*-test or Fisher’s exact test using 2 × 2 contingency tables. * Fisher’s exact test was employed. RA: RA: rheumatoid arthritis, RF: rheumatoid factor, ACPA: anti-citrullinated peptide antibody, DAS: disease activity score, DMARD: disease modifying anti rheumatic drug, csDMARD: conventional synthetic DMARD, bDMARD: biological DMARD, tsDMARD targeted synthetic DMARD.

**Table 2:** Anti-SARS-CoV-2 Abs of RA patients and controls.

	RA	Controls (Early Collection)	*p*	Controls (Late Collection)	*p*
Number	101	117		117	
Anti-SARS-CoV-2 S Ab, U/mL (SD)	324.1 (591.8)	1216.6 (854.4)	<0.0001	582.0 (415.6)	0.0002
Anti-SARS-CoV-2 S Ab positive, *n* (%)	97 (96.0)	116 (100.0)	* 0.0454	116 (100.0)	* 0.0454
Anti-SARS-CoV-2 neutralizing Ab, inhibition rate, % (SD)	10.7 (4.9)	8.6 (6.6)	0.0072	3.1 (3.7)	<0.0001
Anti-SARS-CoV-2 neutralizing Ab, positive, *n* (%)	40 (39.6)	33 (28.4)	* 0.0865	5 (4.3)	* <0.0001
Interval between last vaccination and serum collection, days (SD)	142.1 (31.6)	98.3 (11.2)	<0.0001	257.3 (11.2)	<0.0001

Numbers or average values for each group are shown. Standard deviations or percentages are shown in parentheses. Significance of differences was tested in comparison with the controls (early and late collection of sera) by Student’s *t*-test or Fisher’s exact test using 2 × 2 contingency tables. * Fisher’s exact test was employed. SARS-CoV-2: severe acute respiratory syndrome coronavirus 2, S: spike, RA: rheumatoid arthritis.

**Table 3:** Correlations of clinical manifestations and anti-SARS-CoV-2 Abs in RA.

	Anti-SARS-CoV-2 S Abs			Anti-SARS-CoV-2 Neutralizing Abs		
Clinical Manifestations	PRC	95%CI	*p*	PRC	95%CI	*p*
Age, years	−18.20	(−28.47~−7.92)	0.0007	0.003	(−0.006~0.013)	0.4560
Male	−200.26	(−474.40~73.87)	0.1504	0.11	(−0.13~0.34)	0.3639
Age at onset, years	−6.87	(−13.98~0.23)	0.0579	0.003	(−0.003~0.009)	0.3654
Steinbrocker stage	−50.54	(−143.42~42.35)	0.2829	−0.02	(−0.10~0.06)	0.5847
Steinbrocker class	−78.33	(−239.15~82.49)	0.3362	−0.10	(−0.24~0.03)	0.1313
Body mass index, kg/m^2^	3.39	(−27.17~33.94)	0.8263	0.003	(−0.022~0.028)	0.8215
Smoker or past smoker	−110.37	(−358.74~138.01)	0.3800	0.03	(−0.18~0.24)	0.7623
RF, IU/mL	0.06	(−0.28~0.41)	0.7199	−0.0003	(−0.0006~0.0000)	0.0390
ACPA, IU/mL	0.15	(−0.34~0.64)	0.5481	−0.00005	(−0.00046~0.00035)	0.7934
DAS28	15.55	(−146.63~177.73)	0.8494	−0.11	(−0.24~0.02)	0.1032
DAS28-CRP	68.06	(−104.02~240.13)	0.4343	0.004	(−0.138~0.146)	0.9577
Corticosteroid administration	−215.14	(−451.09~20.81)	0.0735	−0.09	(−0.29~0.11)	0.3944
csDMARDs administration	206.16	(−36.23~448.54)	0.0946	0.06	(−0.14~0.27)	0.5329
bDMARDs administration	−207.75	(−541.41~125.90)	0.2196	−0.38	(−0.65~−0.10)	0.0071
tsDMARDs administration	56.25	(−212.71~325.21)	0.6791	−0.10	(−0.33~0.12)	0.3752
Interval between last vaccination and serum collection, days	4.21	(0.62~7.80)	0.0222	−0.003	(−0.006~0.000)	0.0568

Significance of associations with anti-SARS-CoV-2 S Abs was tested by linear regression analysis. SARS-CoV-2: severe acute respiratory syndrome coronavirus 2, S: spike, RA: rheumatoid arthritis, PRC: partial regression coefficient, CI: confidence interval, RF: rheumatoid factor, ACPA: anti-citrullinated peptide antibody, DAS: disease activity score, DMARD: disease modifying anti rheumatic drug, csDMARD: conventional synthetic DMARD, bDMARD: biological DMARD, tsDMARD targeted synthetic DMARD.

## Data Availability

All data are presented in the paper.

## Supplementary Materials

The following supporting information can be downloaded at: https://www.mdpi.com/article/10.3390/vaccines10081365/s1, Table S1: Correlations of clinical manifestations and anti-SARS-CoV-2 Abs in controls, Table S2: Multiple linear regression analysis of clinical manifestations for anti-SARS-CoV-2 Abs.
