# Supplementary material for: Anti-SARS-CoV-2 Spike Antibody Titers and Neutralizing Antibodies in Vaccinated Rheumatoid Arthritis Patients
Source: Vaccines (Basel). 2022 Aug 21;10(8):1365. doi: 10.3390/vaccines10081365 (PMC9414304; doi:10.3390/vaccines10081365)
Supplement: Supplementary file 1 [file vaccines-10-01365-s001.zip › vaccines-1836667-supplementary.pdf]

Supplementary Table S1. Correlations of clinical manifestations and anti-SARS-CoV-2 Abs in controls.

	Controls (early collection)						Controls (late collection)					
	anti-SARS-CoV-2 S Abs			anti-SARS-CoV-2 neutralizing Abs			anti-SARS-CoV-2 S Abs			anti-SARS-CoV-2 neutralizing Abs		
clinical manifestations	PRC	95%CI	*P*	PRC	95%CI	*P*	PRC	95%CI	*P*	PRC	95%CI	*P*
Age, years	-28.39	(-39.84〜-16.94)	<0.0001	-0.09	(-0.18〜0.01)	0.0710	-12.35	(-18.04〜-6.67)	<0.0001	0.22	(-0.41〜0.84)	0.4935
Male	-420.38	(-745.24〜-95.52)	0.0117	-1.60	(-4.15〜0.94)	0.2145	-167.12	(-326.64〜-7.60)	0.0402	1.16	(-0.29〜2.60)	0.1161
Interval between last vaccination and blood collection, days	-14.11	(-28.01〜-0.21)	0.0467	-0.07	(-0.18〜0.04)	0.1991	-2.96	(-9.82〜3.90)	0.3941	-0.07	(-0.13〜-0.01)	0.0318

Association for anti-SARS-CoV-2 S Abs was tested by linear regression analysis. PRC: partial regression coefficient, CI: confidence interval.

Supplementary Table S2. Multiple linear regression analysis of clinical manifestations for anti-SARS-CoV-2 Abs.

anti-SARS-CoV-2 S Abs	Unconditioned			Conditioned on the other clinical manifestations		
clinical manifestations	PRC	95%CI	*P*	$PRC_{adjusted}$	95%CI	$P_{adjusted}$
Age, years	-26.97	(-31.62～-22.33)	<0.0001	-22.86	(-31.02～-14.71)	<0.0001
Male	-206.75	(-462.47～48.98)	0.1125	-163.02	(-375.53～49.50)	0.1320
Interval between last vaccination and blood collection, days	-8.78	(-12.25～-5.31)	<0.0001	2.46	(-1.51～6.44)	0.2234
RA	-902.16	(-1,101.08～-703.25)	<0.0001	-300.15	(-663.28～62.98)	0.1047

anti-SARS-CoV-2 neutralizing Abs	Unconditioned			Conditioned on the other clinical manifestations		
clinical manifestations	PRC	95%CI	*P*	$PRC_{adjusted}$	95%CI	$P_{adjusted}$
Age, years	0.03	(-0.01～0.07)	0.1715	-0.03	(-0.10～0.03)	0.3213
Male	-1.18	(-2.93～0.57)	0.1856	-0.59	(-2.37～1.19)	0.5124
Interval between last vaccination and blood collection, days	-0.005	(-0.03～0.02)	0.7103	-0.05	(-0.09～-0.02)	0.0022
RA	2.15	(0.59～3.71)	0.0072	5.48	(2.44～8.52)	0.0005

RA: rheumatoid arthritis, PRC: partial regression coefficient, CI: confidence interval. *P*, PRC, 95%CI, $P_{adjusted}$, $PRC_{adjusted}$ were calculated by linear regression analysis on RA patients and controls (early collection).
